# The Power of ECG in Semi-Automated Seizure Detection in Addition to Two-Channel behind-the-Ear EEG

**DOI:** 10.3390/bioengineering10040491

**Published:** 2023-04-20

**Authors:** Miguel Bhagubai, Kaat Vandecasteele, Lauren Swinnen, Jaiver Macea, Christos Chatzichristos, Maarten De Vos, Wim Van Paesschen

**Affiliations:** 1 Department of Electrical Engineering (ESAT), STADIUS Center for Dynamical Systems, Signal Processing and Data Analytics, KU Leuven, 3001 Leuven, Belgium; 2 Laboratory for Epilepsy Research, University Hospital Leuven, 3000 Leuven, Belgium; 3 Department of Development and Regeneration, KU Leuven, 3000 Leuven, Belgium

**Keywords:** epilepsy, seizure detection, multimodal, behind-the-ear EEG, ECG, ictal heart rate

## Abstract

Long-term home monitoring of people living with epilepsy cannot be achieved using the standard full-scalp electroencephalography (EEG) coupled with video. Wearable seizure detection devices, such as behind-the-ear EEG (bte-EEG), offer an unobtrusive method for ambulatory follow-up of this population. Combining bte-EEG with electrocardiography (ECG) can enhance automated seizure detection performance. However, such frameworks produce high false alarm rates, making visual review necessary. This study aimed to evaluate a semi-automated multimodal wearable seizure detection framework using bte-EEG and ECG. Using the SeizeIT1 dataset of 42 patients with focal epilepsy, an automated multimodal seizure detection algorithm was used to produce seizure alarms. Two reviewers evaluated the algorithm’s detections twice: (1) using only bte-EEG data and (2) using bte-EEG, ECG, and heart rate signals. The readers achieved a mean sensitivity of 59.1% in the bte-EEG visual experiment, with a false detection rate of 6.5 false detections per day. Adding ECG resulted in a higher mean sensitivity (62.2%) and a largely reduced false detection rate (mean of 2.4 false detections per day), as well as an increased inter-rater agreement. The multimodal framework allows for efficient review time, making it beneficial for both clinicians and patients.

## 1. Introduction

Epilepsy is one of the most common neurological diseases, affecting approximately 50 million people worldwide [1]. Anti-seizure medications are the first treatment option; however, up to 30% of patients can have refractory epilepsy and require other treatment approaches, such as neurostimulation, ketogenic diet, or surgical procedures [2,3]. For people living with epilepsy, seizure unpredictability increases the risk death and significantly affects their quality of life [4,5]. Conversely, caregivers aim to decrease those risks and improve patients’ health through an expeditious diagnosis and appropriate treatment. Nevertheless, there is a limited list of diagnostic tools when considering long-term follow-up and monitoring treatment response in clinical trials or outpatient scenarios. Current seizure detections and counts are based on expensive in-hospital video-electroencephalography (vEEG), short-term (video) EEG monitoring at home, or seizure diaries, the latter being the primary tool [6,7]. The major disadvantage of seizure diaries is their low sensitivity. For instance, a study of patients with focal epilepsy showed that they reported less than 50% of seizures while admitted for vEEG monitoring [8]. Therefore, accurate seizure detection and counting are crucial during follow-up outside specialized environments [9].

For a long time, machine learning (ML) methods have been deployed to analyze full-scalp EEG data and develop classifiers in order to detect seizures [10]. Researchers have investigated different types of methodologies, such as feature-based [11,12,13,14] and deep learning [15,16,17,18,19] methods. More recently, wearable seizure detection devices have been used to detect focal seizures in a hospital environment using ML methodologies. Vandecasteele et al. developed an automated seizure detection algorithm based on behind-the-ear EEG (bte-EEG) [20], achieving sensitivities of 64.1%, with 2.8 false detections per 24 h. This work was developed on a large and continuous dataset containing recordings of patients with focal epilepsy, mostly originating from the temporal lobe. The results achieved by the algorithm were comparable to visual inspection of the bte-EEG by neurologists, reaching a detection sensitivity of 65.7%. In a more recent work, a multimodal (bte-EEG plus electrocardiography (ECG)) seizure detection framework was proposed for improving the classification performance of seizures in the same dataset [21]. It is known that focal seizures can affect the autonomic nervous system, in particular, the cardiovascular system [22,23]. Therefore, ECG can be used to detect seizures in a subgroup of patients with ictal tachycardia [24]. In [21], the authors implemented a late integration technique for fusing the classification output of a bte-EEG-based algorithm with a single-lead ECG-based seizure detector, to maximize the sensitivity. The addition of ECG data resulted in an increase of 13% in sensitivity (from 79% to 92%). However, the false alarm rate increased from 1 to 1.85 false detections per hour. Despite the high number of false detections, it is of value to implement such framework into clinical practice.

Since annotation and review of EEG takes a long time, it is unfeasible for clinicians to go through the large amounts of data generated when patients are monitored with the wearable devices outside the hospital for several weeks. The use of such automated algorithms for detecting seizures can significantly reduce the amount of data neurologists have to review and consequently provide more significant insights on the progression of the disease compared with the traditional seizure diaries. This framework involves the use of the algorithm as a decision–support system where experts review the possible seizure alarms automatically extracted from the wearable data. The usability of a hybrid automated–visual epileptic patient monitoring was previously studied in patients with absence epilepsy [25]. Here, it was shown that there was a clear benefit in using the hybrid approach, where the classification F1-score of this method was higher (0.87) than visual annotation of the wearable data (0.73) and self-reported documentation (0.15). Additionally, the review time was lower when using the automated annotations for 24 h of bte-EEG recordings (from 1–2 h without versus 5–10 min with automated annotations).

This Phase 1 study, according to the proposed standards for testing seizure detection devices [26], investigates the usability of a multimodal bte-EEG and ECG-based seizure detection algorithm. The main goal is to evaluate the automated decision–support system for visual focal seizure recognition during review. Additionally, we investigate whether ECG and heart rate data affect the readers’ decision when reviewing the automated annotations generated by the algorithm.

By applying this semi-automated approach, we show that it is possible to significantly reduce the review time of the wearable EEG data compared with the traditional annotation method. Additionally, we present evidence of the improvement in classification performance when adding ECG and heart rate to the visual inspection while maintaining the short review time compared with annotating only wearable EEG.

## 2. Materials and Methods

### 2.1. Dataset

The present study was performed on the SeizeIT1 dataset. The data were collected between 23 January 2017, and 26 October 2018, during which 82 patients with refractory focal epilepsy were monitored at the University Hospital Leuven (UZL), Leuven, Belgium. Patients were measured using the 10–20 EEG system, a single-lead ECG placed on the chest and using simultaneous video recording. Four additional Ag/AgCl electrodes were attached behind the ear, on the mastoid bone [20]. Of these patients, only 42 were included in this work. Patients were excluded from the analysis if they had no focal seizure occurrences, data were unreadable (due to strong artifacts), or the wearable modalities were not included in the measurements. For this study, we selected 221 seizures, which were captured during 5284 h of recording. From the seizures recorded, 173 seizures (78.3%) were focal impaired awareness (FIA), 27 (12.2%) were focal aware (FA), 1 (0.5%) was focal to bilateral tonic–clonic (F-BTC), and 20 (9.0%) had unclear classification. Regarding the localization of the seizures recorded, 134 were temporal seizures, 27 fronto-temporal, 15 frontal, 9 occipito-temporal, 2 fronto-parietal, 2 parietal, and 32 had an unclear localization source. The dataset was annotated by a certified epileptologist (W.V.P.) on the basis of the video and 25-channel scalp-EEG data. From all seizures, 176 (80%) had ictal changes in the full-scalp EEG. From the selected patients, 6 had no ictal tachycardia, 23 had ictal tachycardia, defined as an increase in heart rate of at least 20 beats per minute (BPM), and 13 had an increase in heart rate less than 20 BPM. More details regarding the dataset used can be found in Table 1. The ethical committee of UZL and KU Leuven approved the study, and all patients signed an informed consent form for their participation.

### 2.2. Automated Seizure Detection

The seizure detection framework is composed of separate classifiers for bte-EEG and ECG data.

The bte-EEG-based algorithm is composed of a feature extraction and a classification step. The signal is firstly segmented into 2 s windows with 1 s overlap. Subsequently, various time and frequency domain, entropy derived, and asymmetry features are calculated and fed onto the classifier. In total, 67 features were derived from the bte-EEG channels. The classifier is a Support Vector Machine (SVM) with a radial basis function kernel. The bte-EEG algorithm’s detections were post-processed in order to discard possible artifactual segments. Here, a seizure detection was considered valid if at least 8 positive alarms were present in 10 consecutive 2 s segments (with a 1 s overlap); thus, the minimum duration of an automated detection is 10 s. A full detailed description of the bte-EEG-based algorithm can be found in [20].

Similarly, the ECG-based algorithm is composed of a feature extraction and classification modules [27]. The ECG features are extracted from the heart rate information, derived from an ensemble of R-peak detection algorithms [24,28,29]. In contrast to the EEG-based classifier, the ECG signal is segmented in windows of 60 s, with a 10 s overlap. Furthermore, the seizure annotations for the ECG data were extended in time by 30 s before and after to train the algorithm to cope with the slower ictal patterns present on the cardiac activity measured [30]. The ECG-based algorithm produced seizure detections of at least 60 s.

**Figure 1:**
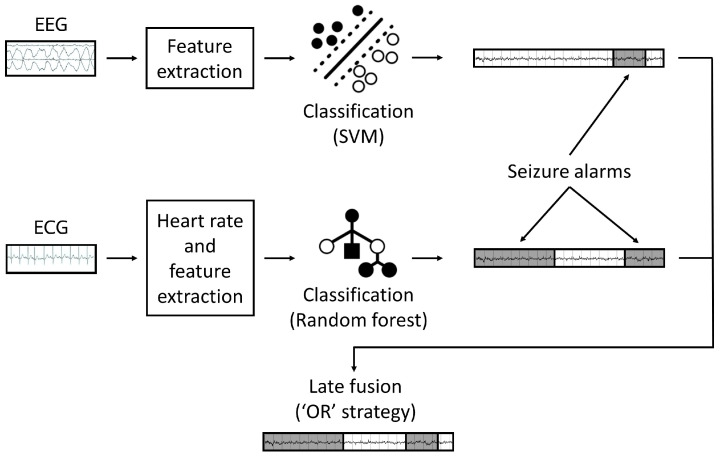
Graphical representation of the multimodal automated multimodal seizure detection framework based on bte-EEG and ECG.

The multimodal framework employs a late fusion technique with an ‘OR’ strategy [21]. Here, the classification output of each modality is integrated in a way that a seizure detection is counted if an alarm is present in either the bte-EEG or ECG, focusing on achieving high sensitivity. The output of the multimodal algorithm is composed of a union of the unimodal algorithms’ detections and, in the case of an overlapping alarms, the bte-EEG detection segment was taken. Figure 1 shows a graphical representation of the complete multimodal framework.

### 2.3. Semi-Automated Seizure Detection

#### 2.3.1. Data Preparation

The bte-EEG, raw ECG, and heart rate data were collected and saved in European Data Format (EDF) format, along with the ground-truth seizure annotations. Additionally, all the automated seizure detections’ start and end times were included in the files. In the case of correct detections, the ground-truth annotations were substituted by the automated seizure detections.

#### 2.3.2. Visual Experiment

**Figure 2:**
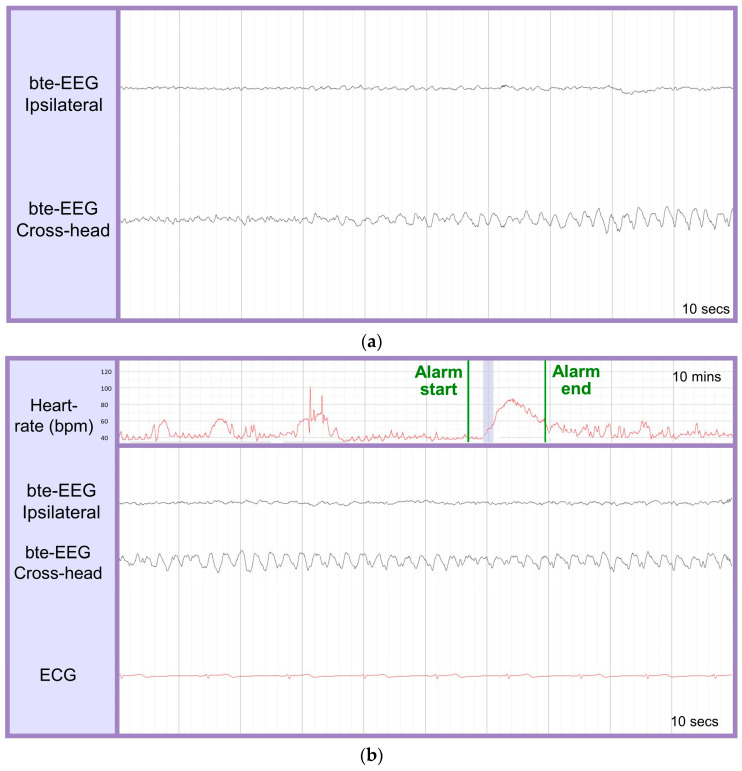
Presentation setup for the two visual experiments: (**a**) setup for visual annotation of the bte-EEG data; (**b**) setup for visual annotation of the bte-EEG and ECG data. In (**a**,**b**), the black time-series corresponds to the ipsilateral (top) and cross-head (bottom) bte-EEG channel. In (**b**), the top red signal corresponds to the heart rate derived from ECG, and the bottom red signal shows the raw ECG data. The heart rate data are presented in a 10 min window, and the other signals in a 10 s window. The green vertical lines indicate the start and end of the detected seizure, and the purple shaded area on the heart rate plot depicts the 10 s window presented in the bte-EEG and ECG plot.

The EEG and ECG data were visualized with BrainRT (O.S.G., Kontich, Belgium). Two readers, one neurologist (JM) and one biomedical clinical data expert (LS), were presented with the prepared data files, blinded to any clinical information. The readers were instructed to inspect the seizure detections indicated in the recordings twice, with two different visual setups:

1. Two-channel bte-EEG (Figure 2a);
2. Two-channel bte-EEG with ECG and additional graph plotting the extracted heart rate (Figure 2b).

The two-channel EEG montage was chosen instead of the three-channel montage that was used in previous studies [20,21] to simulate the wearable Sensor Dot from Byteflies [31], which is in the process of validation on the second iteration of the SeizeIT project [32]. The two channels are composed of one cross-head and one ipsilateral channel, measured on the hemisphere of the seizure onset. The bte-EEG data were processed with a 35 Hz low-pass filter and a 0.5 Hz high-pass filter and were presented within an amplitude scale of 70 µV/cm. The visualization window was set to 10 s, and the heart rate data were presented within a 10 min window. Readers were allowed to scroll freely within the recording. For each marked seizure predicted by the algorithm, the readers relabeled the data segments as seizure, artifact, or other physiological event. In addition, they had to record the review time.

#### 2.3.3. Performance Evaluation

To evaluate the performance of the automated algorithm, the seizure detections were compared with the ground-truth annotations from the vEEG. In case of the visual experiment, the relabeled segments were compared with only the automatically generated annotations by the algorithm.

For the algorithm and both visual experiments, 4 different metrics were calculated on the basis of the true positives (TP), false positives (FP), and false negatives (FN):

- Sensitivity: TP/(TP + FN)
- False detection rate per 24 h (FD/24 h): 3600 × 24 × FP/D_recordings_, where D is the duration of the recordings in seconds
- Positive predictive value (PPV): TP/(TP + FP)
- F1-score: 2 × (Sensitivity × PPV)/(Sensitivity + PPV)

For the algorithmic performance evaluation, a TP corresponds to an overlap between the ground-truth and the seizure detection; an FP is counted when a detection does not overlap with a true seizure annotation; and an FN is counted when the automated algorithm does not detect a true seizure. In the case of the visual experiments, a TP indicates that the reader was able to correctly classify a seizure detection when it overlaps with a ground-truth seizure annotation; an FP involves classifying a detection as seizure when it does not overlap with a ground-truth annotation; and an FN when a correct detection is not classified as a seizure by the reader. The false negatives of the algorithm were not taken into account for the performance metrics of the visual experiment to simulate a real-case scenario of the semi-automated seizure detection framework.

In addition to the listed performance metrics, the inter-rater variability was assessed by calculating the Cohen’s kappa coefficient [33] between the two raters.

## 3. Results

In total, there were 7975 seizure detections (including TPs (*n* = 230), FPs (*n* = 7721), and FNs (n = 24) of the algorithm). The difference (n = 9) in the number of correct detections and the 221 seizures recorded is due to multiple detections of the same seizure. The defined metrics in the method section are presented in Table 2.

The automated seizure detection algorithm was able to detect 196 of the 221 seizures, resulting in a high sensitivity of 90.5%. However, the false detection rate was substantially high at approximately 43 FD/24 h. This negatively affected the PPV and the F1-score, both at very low values.

Regarding the bte-EEG visual experiment, the readers obtained a mean detection sensitivity of 59.1%, with 6.5 FD/24 h, a PPV of 13.4%, and an F1-score of 0.2. Adding ECG and heart rate data to the visual detection of seizures improved the readers’ performance. The mean sensitivity was increased to 62.2%, and the false detection rate was substantially lower at 2.4 FD/24 h. Both readers improved the PPV and F1-score when annotating the detections with the added ECG and heart rate data (mean of 25.7% and 0.4, respectively). The difference in the readers’ performance between the two experimental conditions was tested with McNemar’s test. The visual review performance was significantly better when both bte-EEG and ECG data were presented (*p*-value of 0.00). In both visual setups, the readers reported the time to review one day’s worth of data as approximately 8 min by visualizing only the detections provided by the algorithm. There was a small decrease when ECG data were available. The readers were also instructed to blindly annotate seizures on a full day’s worth of one bte-EEG recording, without video or full-scalp EEG data and with no assistance from the algorithm. The reported time to review was approximately 56 min.

**Figure 3:**
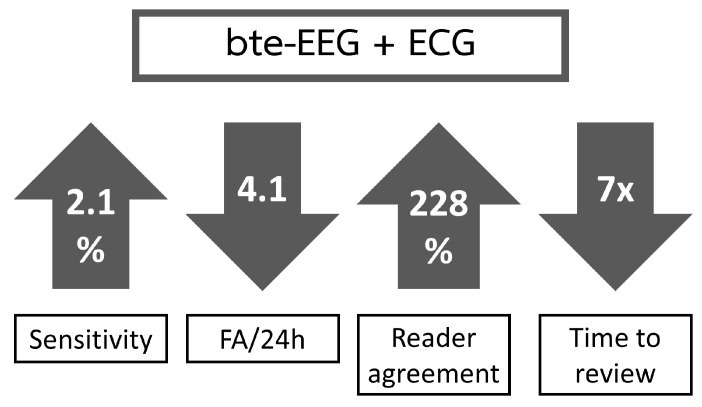
Diagram representing the gain in sensitivity, inter-rater agreement, and reduction of false alarms by reviewing the data with the semi-automated multimodal framework (bte-EEG and ECG) compared with the unimodal framework (bte-EEG). The reduction in the time to review is compared with the reported time to review 24 h of bte-EEG recordings without automated assistance.

The inter-rater reliability was quantified using Cohen’s kappa. From Table 3, we can conclude that the agreement between the two readers was fair (0.21) when visualizing only bte-EEG for validation of the seizure detections. The addition of the ECG and heart rate data to the analysis resulted in a better agreement between the raters, increasing the kappa coefficient to 0.48. The change in the most significant performance metrics when ECG and heart rate data is added to the visual review is presented in Figure 3.

**Figure 4:**
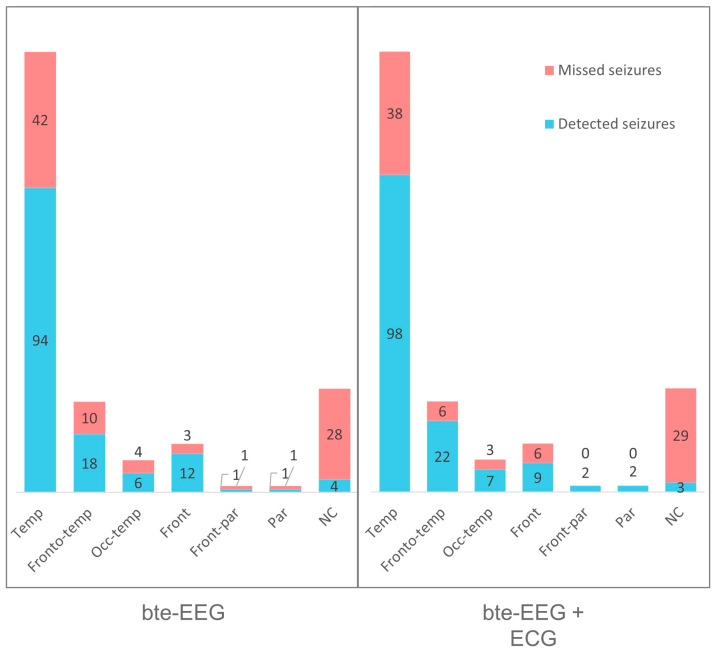
Number of detected (correctly annotated) and missed (mislabeled) seizures of the two readers in the two experimental setups readers, discriminated by the location of the seizure onset (Temp—Temporal, Fronto-temp—Fronto-temporal, Occ-temp—Occipital-temporal, Front—Frontal, Fronto-par—Fronto-parietal, Par—Parietal, or NC—Not Clear).

The readers correctly annotated 136 alarms in the first experimental setup and 143 in the second. Figure 4 discriminates the number of detected and missed seizures by the two readers in the two experiments for each seizure onset location in the dataset. The number of detected seizures is higher in the bte-EEG and ECG setup for all types except frontal and non-clear seizure onset locations. The highest number of mislabeled seizure alarms corresponds to temporal (42 and 38) and non-clear (28 and 29) seizure localizations (in the bte-EEG setup and bte-EEG and ECG setup, respectively).

**Figure 5:**
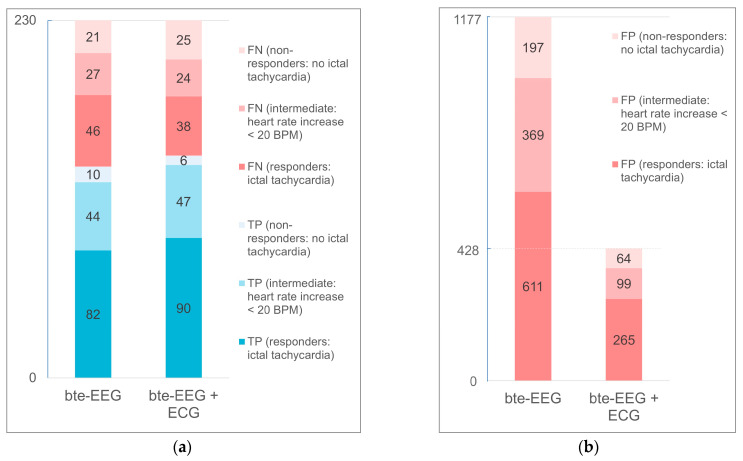
(**a**) Number of true positives (TP, correctly annotated seizures) and false negatives (FN, mislabeled seizure segments) and (**b**) number of false positives (FP, detections incorrectly classified as seizures) of the two readers in the two experimental setups readers discriminated by the type of patient (responder, non-responder, or intermediate response) according to the presence of ictal ECG changes.

When considering the patient’s status in terms of having ictal heart rate changes, we can see that using ECG data improves the detection sensitivity of the responders (patients with ictal tachycardia or an increase in heart rate of more than 20 BPM was observed). From Figure 5a, both readers correctly annotated more seizures of responder patients when ECG and heart rate data were present along with the bte-EEG (mean increase of 8 seizures detected). In addition, the readers achieved better sensitivity for patients who had an intermediate tachycardia effect (increase in heart rate of less than 20 BPM) during the seizure. Both readers mislabeled a mean of five more seizure detections in the non-responders group when ECG data were shown. Regarding the false detections, the number of alarms incorrectly classified as seizures by the readers decreased substantially in patients of all statuses. Figure 5b depicts the number of FPs annotated by the readers in each experimental setup. In the bte-EEG only experimental setup, a mean of 1177 FPs was reported, whereas in the bte-EEG and ECG review, the readers achieved a mean of 428 false detections. The difference in the number of FPs between experimental setups was higher in patients who had ictal tachycardia (mean difference of 346).

**Figure 6:**
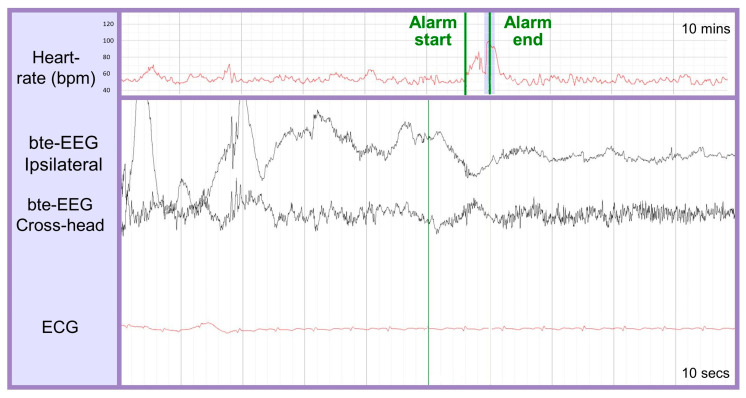
Example of a detected seizure segment mislabeled as a non-seizure on the bte-EEG experimental setup but corrected on the bte-EEG and ECG and heart rate setup (change from FN to TP). The top plot contains the heart rate data in red within a 10 min window, and the bottom contains the bte-EEG channels (in black) and ECG data (in red) within a 10 s window. The shaded section of the heart rate plot corresponds to the plotted area of the bottom one. The green vertical lines indicate the automated algorithm’s time events.

**Figure 7:**
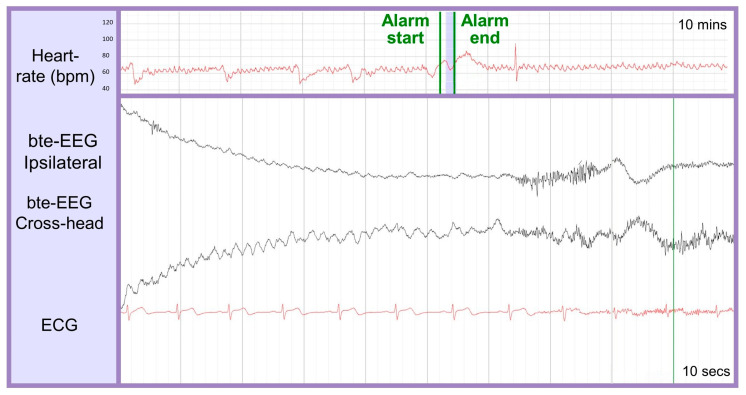
Example of a detected seizure segment correctly labeled by the readers on the bte-EEG experimental setup but mislabeled on the bte-EEG and ECG and heart rate setup (change from TP to FN). The top plot contains the heart rate data in red within a 10 min window, and the bottom contains the bte-EEG channels (in black) and ECG data (in red) within a 10 s window. The shaded section of the heart rate plot corresponds to the plotted area of the bottom one. The green vertical lines indicate the automated algorithm’s time events.

**Figure 8:**
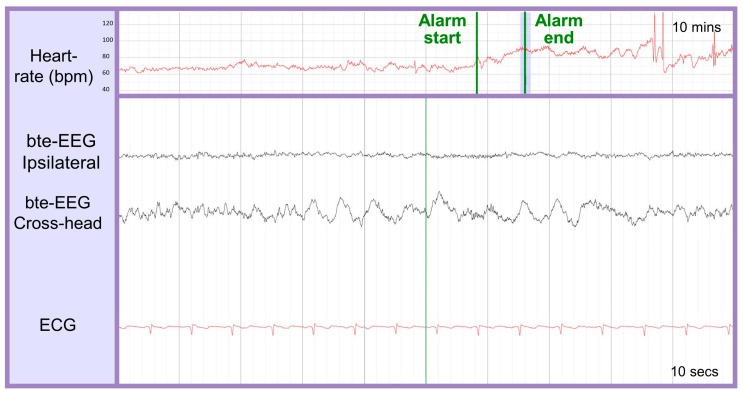
Example of a detected segment incorrectly labeled as a seizure by the algorithm and by the readers on both the bte-EEG setup and the bte-EEG and ECG and heart rate setups(FP). The top plot contains the heart rate data in red within a 10 min window, and the bottom contains the bte-EEG channels (in black) and ECG data (in red) within a 10 s window. The shaded section of the heart rate plot corresponds to the plotted area of the bottom one. The green vertical lines indicate the automated algorithm’s time events.

Examples of correctly annotated seizures based on bte-EEG data only and on both bte-EEG and ECG with heart rate data are visualized in Figure 2. It is possible to see the typical ictal rhythmic pattern in the cross-lateral bte-EEG channel. In Figure 2b, the plotted section shows a sudden increase in heart rate overlapping the seizure segment. Figure 6 shows an example of a detected seizure of a patient with ictal tachycardia that was annotated as a non-seizure within the first experimental setup, but correctly classified by both readers on the second experimental setup. In this case, the seizure is not clearly visible on the bte-EEG signal but is accompanied with a heart rate increase. An example of patient’s seizure without tachycardia is shown in Figure 7. On the bte-EEG setup, the readers correctly classified this particular segment as a seizure. However, when heart rate data were presented, the readers discarded the detected segment since the heart rate did not show a significant increase. Figure 8 depicts a false detection in both experimental setups. The heart rate plot shows a significant increase (more than 20 BPM) simultaneously with a presence of rhythmic artifactual oscillations in the bte-EEG signal.

## 4. Discussion

In this study, we investigated the usability of an automated seizure detection framework based on bte-EEG and ECG as a decision–support tool for monitoring patients with epilepsy. Focal seizures are the most prevalent seizure type, accounting for 60% of cases of adult epilepsy [34]. Detecting focal seizures with wearable EEG setups is more challenging compared with generalized seizures due to the heterogeneity of EEG patterns and the fact that some seizures become invisible on reduced montage EEGs [35]. Wearable EEG setups are developed with the intention of causing minimal obtrusion to the patients. In this sense, the number of electrodes used is significantly reduced, and their placement is usually on non-standard locations of the scalp. Unlike generalized seizures, the onset location of focal epilepsy is more challenging to capture by the wearable EEG because it is strongly dependent on the electrode–onset distance and propagation pathways [36,37]. Despite this, we recommended using a combination of electrodes that allow ipsilateral (based on the onset hemisphere) and cross-head channel montages to detect focal seizures [38].

Other studies have evaluated the utility of reduced EEG montage setups for visual seizure detection in the case of focal seizures. In [39], an EasyCap EEG system with reduced number of channels was used, achieving 39.2% detection sensitivity where only 31% of focal seizures recorded were correctly identified. A single-channel wearable EEG device from Epilog [40] was evaluated in [41], where epileptologists obtained a sensitivity of 55% for focal impaired awareness seizures.

In the first experimental setup of this work, the readers achieved similar performances when evaluating the automatically generated seizure detections (sensitivity of 59.1%). In a real-use case scenario, reviewing 24 h of bte-EEG with the assisted monitoring framework allowed a 7-fold reduction in review time when compared with the estimates reported by the annotators for blindly analyzing the same data without the algorithm’s alarms (8 min versus 56 min, respectively). The non-assisted bte-EEG review time is similar to reviewing full-scalp vEEG data, according to the literature [25]. Despite the number of electrodes in the wearable EEG setup being much less than a standard full-scalp EEG setup, readers required more attention to identify seizures in the reduced channel montage. The false detection rate was considerably lower than the automated detection. In [20], the authors reported a sensitivity of 65.7% for detecting focal seizures in bte-EEG. Here, the epileptologist was presented with 10-minute-long data segments containing seizures and background wearable EEG. Considering the setup and electrode placement, it is expected that seizures with onset on the temporal and fronto-temporal lobes are more likely to be captured in the wearable recordings. The dataset used in this study contained a majority of temporal lobe seizures. However, 38 temporal lobe seizures were missed by the readers in the multimodal setup. The main reason for missing ictal events was the presence of muscle artifacts in the bte-EEG and low impedance from electrode movements. Extratemporal seizures were correctly annotated, indicating the capabilities of the wearable bte-EEG capturing ictal patterns from more distant cerebral regions. In seizures classified as having non-clear onset localization, readers could not identify ictal patterns in the given alarms. In these cases, the subtle ictal changes in the signal were marked as artifacts or other physiological events. Contrary to a vEEG setup, the readers blindly annotated the bte-EEG and ECG. Without the support of video data, the likelihood of discarding seizure alarms when no clear ictal patterns are visible is higher.

The results show a relatively low agreement between the two raters when annotating only the bte-EEG signals, which is reflected by the calculated 0.20 Cohen’s kappa coefficient. This shows the high inter-rater variability when interpreting reduced montage EEG recordings for recognizing focal seizures. This effect is also visible in other validation studies of wearable EEG setups for seizure detection. In [41], two neurophysiologists annotated full-scalp and in-ear EEG recordings of patients with focal epilepsy, achieving 56% and 92% sensitivity, respectively, on the wearable EEG.

In this study, the sensitivity achieved with the automated algorithm was much higher than visual inspection of the wearable EEG modality. In addition to the higher presence of muscle and other physiological artifacts on wearable EEG setups, the loss in sensitivity by the readers is heavily affected by the standard procedures and rules for seizure detection to which they are subjected. Within the wearable setup, the readers relied on seizure patterns present mainly on the cross-head channel, whereas the spread of focal seizures on full-scalp EEG measurements can be observed on multiple channels. Despite this, the readers were somewhat experienced in reading two-channel EEG, but the level of certainty was significantly lower within this setup. On the other hand, the automated algorithm is based purely on the data patterns used during the training phase. In this sense, it is logical that its sensitivity is higher when tuned for this purpose, with the drawback of producing a high number of false detections due to the presence of artifacts [20]. Considering the automated algorithm as a support tool, the present study confirms its usability, as it can achieve a detection performance close to the theoretical boundary of gold-standard visual seizure assessment.

The use of ECG data was shown to be beneficial for validating the automated seizure detections. When raw ECG and heart rate signals were presented together with bte-EEG data, the detection performance of each reader increased, reflected by the difference in the F1-scores (Reader 1 improved from 0.13 to 0.32 and Reader 2 from 0.28 to 0.40). Figure 5a shows that the increase in sensitivity was due mainly to added detectable seizures of patients with ictal tachycardia, where the TP and FN numbers increased and decreased, respectively, when compared with the first experiment. These findings substantiate the usability of ECG data for focal seizure detection.

The presence of ictal tachycardia provides the basis for the decision of the readers in cases where patients had an increase of more than 20 BPM. The bte-EEG data can be corrupted with artifacts, causing uncertainty on the readers decision. In the example shown in Figure 6, the readers appeared to weight their decisions on the presence of a sudden increase in the patients’ heart rate, correcting their evaluation to a positive seizure detection. In this particular case, the ictal heart rate increase is corrupted with an abrupt drop resulting from artifacts on the ECG signal. Including the raw ECG allows readers to have more information on possible noise segments when analyzing the heart rate and, thus, produce a better decision during review. Both readers misclassified more seizures of patients without tachycardia when the heart rate data were available for review. Seizures corrupted with noise or muscle artifacts can be more dubious when visualizing wearable EEG data. When the expected increase in heart rate is not present, the readers relied more on the second modality, wrongfully discarding some of the detected seizures on the experimental setup 1. An example of a misclassified seizure segment due to an unclear seizure pattern on the bte-EEG data and lack of ictal heart rate increase can be seen in Figure 7. The same negative effect on the sensitivity can occur due to corrupted ECG data. The presence of interferences in the cardiac signal can cause a wrong derivation of the R-R peak intervals and, thus, the heart rate, causing difficulties to the readers in evaluating the detected seizure segments. Other causes for false detections and revisions can be the presence of artifacts due to increased physical effort, where the movement causes a rhythmic seizure-like activity on the bte-EEG data accompanied with a heart rate increase, misleading both the algorithm and readers to believe it is a seizure segment (Figure 8). Despite this, the agreement between the readers increased significantly, represented in the Cohen’s kappa value. The benefits in adding ECG and heart rate data to the review process were noted mainly in the decreased false alarm rate. The reduction of positives occurs mainly in patients with ictal tachycardia. The number of false detections also decreases in patients with unclear or no tachycardia (Figure 5b). The absence of a heart rate increase presented in the visual setup allows the readers to discard most of the bte-EEG false detections caused by artifacts or other physiological events.

In clinical practice, in order to take advantage of the extra modality, it is essential to identify beforehand whether patients have ictal tachycardia, determining whether the use of ECG would benefit the detection performance. The dataset used in this study contained a majority of patients who had ictal tachycardia (53%). Such incidence may not be representative of the full population of epileptic patients. There are reports that seizure type, duration, and conditions of activity affect the occurrence of ictal heart rate changes, but it is often re-occurring in seizure episodes of the same patient [23]. In this sense, it is possible to personalize the diagnostic and the automated algorithm, allowing for further improvements in the automatic seizure detection and the visual review, potentially decreasing review time and increasing sensitivity and confidence within the readers. Using the presented framework in a real-case setting outside of controlled environments and with wearable devices still needs to be evaluated and will introduce more challenges related to signal quality and presence of artifacts.

## 5. Conclusions

The need for automated seizure detection frameworks is of high relevance in clinical practice. We presented a semi-automated multimodal seizure detection methodology based on a two-channel EEG setup and ECG that outperforms standard methods. Due to the high sensitivities achieved, the developed automatic seizure detection algorithms can be used as supporting tools for visual review of focal seizures. The main drawbacks of using such modalities are the difficulties in capturing ictal patterns and the susceptibility to interferences and artifactual data, causing lower confidence in the detected seizure segments and high inter-rater variability. Adding ECG and heart rate data to the bte-EEG when reviewing the automated detections increases inter-rater agreement and reduces the high number of false detections produced by the algorithm. The addition of heart rate data for visual seizure detection is relevant in patients with ictal tachycardia.

## Figures and Tables

**Table 1:** Overview of the dataset used in the study (SeizeIT1). The table shows the number of seizures, seizure types (FA—Focal Aware, FIA—Focal Impaired Awareness, F-BTC—Focal to Bilateral Tonic–Clonic, or NC—Not Clear), localization (Temp—Temporal, Par—Parietal, Fronto-temp—Fronto-temporal, Fronto-par—Fronto-parietal, Occipito-temp—Occipito-temporal, or NC—Not Clear), lateralization (L—Left, R—Right, bi—bilateral, or NC—Not Clear), and presence of ictal tachycardia.

Patient	Nr. Seizures	Seizure Type	Localization	Lateralization	Ictal Tachycardia
**1**	6	FIA	Temp	R (1) and bi (5)	Yes
**2**	9	FIA	Fronto-temp	R	Yes
**3**	2	FIA	Temp	R	Yes
**4**	8	FIA	Temp	R	Yes
**5**	3	FIA	Temp	L	No
**6**	2	FIA	Temp	L	Yes
**7**	17	NC	NC	NC	Yes
**8**	2	FIA	Temp	R	Intermediate
**9**	2	FA	NC	NC	No
**10**	2	FIA	Fronto-par	R (1) and bi (1)	Yes
**11**	5	FIA	Temp	L	Intermediate
**12**	6	FIA	Temp	L	Intermediate
**13**	5	FIA	Temp (3) and NC (2)	R (3) and NC (2)	Intermediate
**14**	2	FIA	Temp	L	Yes
**15**	2	FA (1) and FIA (1)	Temp (1) and NC (1)	R (1) and NC (1)	No
**16**	2	FA	Temp (1) and NC (1)	R (1) and NC (1)	Yes
**17**	3	NC	NC	NC	Intermediate
**18**	6	FA	NC	NC	No
**19**	3	FIA	Temp	L	Yes
**20**	5	FIA	Temp	L (3) and R (2)	Intermediate
**21**	15	FIA	Frontal	NC	Yes
**22**	3	FIA	Temp	R	Intermediate
**23**	7	FIA	Temp	L	No
**24**	5	FIA	Temp	L	Yes
**25**	4	FIA	Temp	L	Yes
**26**	22	FA (14) and FIA (8)	Temp (12) and Fronto-temp (10)	L (3) and R (19)	Intermediate
**27**	9	FIA	Temp	L (4), R (3), and bi (2)	No
**28**	3	FA (2) and F-BTC (1)	Temp (1) and Par (2)	R	Yes
**29**	2	FIA	Temp	R	Yes
**30**	3	FIA	Fronto-temp	L	Yes
**31**	2	FIA	Occipito-temp	L	Intermediate
**32**	6	FIA	Temp	R	Intermediate
**33**	8	FIA	Temp (1) and Occipito-temp (7)	R	Yes
**34**	5	FIA	Fronto-temp	L	Intermediate
**35**	5	FIA	Temp	L	Yes
**36**	6	FIA	Temp	R	Yes
**37**	5	FIA	Temp	R	Yes
**38**	2	FIA	Temp	L	Intermediate
**39**	2	FIA	Temp	bi	Intermediate
**40**	5	FIA	Temp	R	Yes
**41**	2	FIA	Temp	L	Yes
**42**	8	FIA	Temp	L (3), R (1), and bi (4)	Yes

**Table 2:** Performance metrics of the readers (mean (reader 1–reader 2)) in each experimental setup (visual analysis of only bte-EEG and bte-EEG together with ECG and heart rate data) and the multimodal seizure detection algorithm.

	Sensitivity (%)	FA/24 h	PPV (%)	F1-Score	Time to Review/24 h
Multimodal algorithm	90.6	43.4	2.9	0.06	-
bte-EEG	59.1 (63.0–55.2)	6.5 (10.1–3.0)	13.4 (7.49–19.04)	0.2 (0.1–0.3)	8.9 min (8.4–9.5)
bte-EEG and ECG	62.2 (63.0–61.3)	2.4 (2.9–1.9)	25.7 (21.8–29.7)	0.4 (0.3–0.4)	7.6 min (7.2–8.0)

**Table 3:** Inter-rater variability between the two readers for each experimental setup.

Inter-Rater Variability (Cohen’s Kappa)	
bte-EEG	0.21
bte-EEG and ECG	0.48

## Data Availability

The data presented in this study are available on request from the corresponding author. The data are not publicly available due to privacy restrictions.

## References

[1] World Health Organization Epilepsy: A Public Health Imperative. https://apps.who.int/iris/bitstream/handle/10665/325293/9789241515931-eng.pdf.

[2] Goldenberg M.M. (2010). Overview of Drugs Used for Epilepsy and Seizures: Etiology, Diagnosis, and Treatment. Pharm. Ther..

[3] Laxer K.D., Trinka E., Hirsch L.J., Cendes F., Langfitt J., Delanty N., Resnick T., Benbadis S.R. (2014). The Consequences of Refractory Epilepsy and Its Treatment. Epilepsy Behav..

[4] Sveinsson O., Andersson T., Mattsson P., Carlsson S., Tomson T. (2020). Clinical Risk Factors in SUDEP: A Nationwide Population-Based Case-Control Study. Neurology.

[5] Salas-Puig X., Iniesta M., Abraira L., Puig J., QUIN-GTC study group Accidental Injuries in Patients with Generalized Tonic-Clonic Seizures (2019). A Multicenter, Observational, Cross-Sectional Study (QUIN-GTC Study). Epilepsy Behav..

[6] Shih J.J., Fountain N.B., Herman S.T., Bagic A., Lado F., Arnold S., Zupanc M.L., Riker E., Labiner D.M. (2018). Indications and Methodology for Video-electroencephalographic Studies in the Epilepsy Monitoring Unit. Epilepsia.

[7] Fisher R.S., Blum D.E., DiVentura B., Vannest J., Hixson J.D., Moss R., Herman S.T., Fureman B.E., French J.A. (2012). Seizure Diaries for Clinical Research and Practice: Limitations and Future Prospects. Epilepsy Behav..

[8] Hoppe C., Poepel A., Elger C.E. (2007). Epilepsy: Accuracy of Patient Seizure Counts. Arch. Neurol..

[9] Hubbard I., Beniczky S., Ryvlin P. (2021). The Challenging Path to Developing a Mobile Health Device for Epilepsy: The Current Landscape and Where We Go from Here. Front. Neurol..

[10] Siddiqui M.K., Morales-Menendez R., Huang X., Hussain N. (2020). A Review of Epileptic Seizure Detection Using Machine Learning Classifiers. Brain Inform..

[11] Boonyakitanont P., Lek-uthai A., Chomtho K., Songsiri J. (2020). A Review of Feature Extraction and Performance Evaluation in Epileptic Seizure Detection Using EEG. Biomed. Signal Process. Control.

[12] Aayesha, Qureshi M.B., Afzaal M., Qureshi M.S., Fayaz M. (2021). Machine Learning-Based EEG Signals Classification Model for Epileptic Seizure Detection. Multimed. Tools Appl..

[13] Zarei A., Asl B.M. (2021). Automatic Seizure Detection Using Orthogonal Matching Pursuit, Discrete Wavelet Transform, and Entropy Based Features of EEG Signals. Comput. Biol. Med..

[14] Fraiwan M.A., Alafeef M. (2022). Multiclass Epilepsy Classification Using Wavelet Decomposition, Direct Quadrature, and Shannon Entropy. J. Eng. Sci. Technol..

[15] Roy Y., Banville H., Albuquerque I., Gramfort A., Falk T.H., Faubert J. (2019). Deep Learning-Based Electroencephalography Analysis: A Systematic Review. J. Neural Eng..

[16] Zhou M., Tian C., Cao R., Wang B., Niu Y., Hu T., Guo H., Xiang J. (2018). Epileptic Seizure Detection Based on EEG Signals and CNN. Front. Neuroinform..

[17] Ullah I., Hussain M., Qazi E.-U.-H., Aboalsamh H. (2018). An Automated System for Epilepsy Detection Using EEG Brain Signals Based on Deep Learning Approach. Expert Syst. Appl..

[18] Yuan Y., Xun G., Jia K., Zhang A. (2017). A Multi-View Deep Learning Method for Epileptic Seizure Detection Using Short-Time Fourier Transform. Proceedings of the 8th ACM International Conference on Bioinformatics, Computational Biology, and Health Informatics.

[19] Yuvaraj R., Thomas J., Kluge T., Dauwels J. A Deep Learning Scheme for Automatic Seizure Detection from Long-Term Scalp EEG. Proceedings of the 2018 52nd Asilomar Conference on Signals, Systems, and Computers.

[20] Vandecasteele K., De Cooman T., Dan J., Cleeren E., Van Huffel S., Hunyadi B., Van Paesschen W. (2020). Visual Seizure Annotation and Automated Seizure Detection Using Behind-the-ear Electroencephalographic Channels. Epilepsia.

[21] Vandecasteele K., De Cooman T., Chatzichristos C., Cleeren E., Swinnen L., Ortiz J.M., Van Huffel S., Dümpelmann M., Schulze-Bonhage A., De Vos M. (2021). The Power of ECG in Multimodal Patient-specific Seizure Monitoring: Added Value to an EEG-based Detector Using Limited Channels. Epilepsia.

[22] Sevcencu C., Struijk J.J. (2010). Autonomic Alterations and Cardiac Changes in Epilepsy. Epilepsia.

[23] Zijlmans M., Flanagan D., Gotman J. (2002). Heart Rate Changes and ECG Abnormalities during Epileptic Seizures: Prevalence and Definition of an Objective Clinical Sign. Epilepsia.

[24] Cooman T.D., De Cooman T., Varon C., Hunyadi B., Van Paesschen W., Lagae L., Van Huffel S. (2017). Online Automated Seizure Detection in Temporal Lobe Epilepsy Patients Using Single-Lead ECG. Int. J. Neural Syst..

[25] Swinnen L., Chatzichristos C., Jansen K., Lagae L., Depondt C., Seynaeve L., Vancaester E., Van Dycke A., Macea J., Vandecasteele K. (2021). Accurate Detection of Typical Absence Seizures in Adults and Children Using a Two-channel Electroencephalographic Wearable behind the Ears. Epilepsia.

[26] Beniczky S., Ryvlin P. (2018). Standards for Testing and Clinical Validation of Seizure Detection Devices. Epilepsia.

[27] Vandecasteele K., De Cooman T., Gu Y., Cleeren E., Claes K., Van Paesschen W., Van Huffel S., Hunyadi B. (2017). Automated Epileptic Seizure Detection Based on Wearable ECG and PPG in a Hospital Environment. Sensors.

[28] Li C., Zheng C., Tai C. (1995). Detection of ECG Characteristic Points Using Wavelet Transforms. IEEE Trans. Biomed. Eng..

[29] Varon C., Caicedo A., Testelmans D., Buyse B., Van Huffel S. (2015). A Novel Algorithm for the Automatic Detection of Sleep Apnea From Single-Lead ECG. IEEE Trans. Biomed. Eng..

[30] Varon C., Jansen K., Lagae L., Van Huffel S. (2015). Can ECG Monitoring Identify Seizures?. J. Electrocardiol..

[31] Byteflies. https://byteflies.com/.

[32] Vertes G. SeizeIT2. https://eithealth.eu/project/seizeit2/.

[33] Cohen J. (1968). Weighted Kappa: Nominal Scale Agreement with Provision for Scaled Disagreement or Partial Credit. Psychol. Bull..

[34] Téllez-Zenteno J.F., Hernández-Ronquillo L. (2012). A Review of the Epidemiology of Temporal Lobe Epilepsy. Epilepsy Res. Treat..

[35] Rubin M.N., Jeffery O.J., Fugate J.E., Britton J.W., Cascino G.D., Worrell G.A., Hocker S.E., Wijdicks E.F., Rabinstein A.A. (2014). Efficacy of a Reduced Electroencephalography Electrode Array for Detection of Seizures. Neurohospitalist.

[36] Tacke M., Janson K., Vill K., Heinen F., Gerstl L., Reiter K., Borggraefe I. (2022). Effects of a Reduction of the Number of Electrodes in the EEG Montage on the Number of Identified Seizure Patterns. Sci. Rep..

[37] de Curtis M., Avoli M. (2015). Initiation, Propagation, and Termination of Partial (Focal) Seizures. Cold Spring Harb. Perspect. Med..

[38] Gu Y., Cleeren E., Dan J., Claes K., Van Paesschen W., Van Huffel S., Hunyadi B. (2017). Comparison between Scalp EEG and Behind-the-Ear EEG for Development of a Wearable Seizure Detection System for Patients with Focal Epilepsy. Sensors.

[39] McKenzie E.D., Lim A.S.P., Leung E.C.W., Cole A.J., Lam A.D., Eloyan A., Nirola D.K., Tshering L., Thibert R., Garcia R.Z. (2017). Validation of a Smartphone-Based EEG among People with Epilepsy: A Prospective Study. Sci. Rep..

[40] Epitel. https://www.epitel.com/.

[41] Zibrandtsen I.C., Kidmose P., Christensen C.B., Kjaer T.W. (2017). Ear-EEG Detects Ictal and Interictal Abnormalities in Focal and Generalized Epilepsy—A Comparison with Scalp EEG Monitoring. Clin. Neurophysiol..

